# Gαq/11 aggravates acute lung injury in mice by promoting endoplasmic reticulum stress-mediated NETosis

**DOI:** 10.1186/s10020-025-01118-4

**Published:** 2025-02-19

**Authors:** Qian Xiang, Yang Tian, Kai Yang, Yaqin Du, Jian Xie

**Affiliations:** 1 Department of Pharmacology, School of Basic Medical Sciences, State Key Laboratory of Vascular Homeostasis and Remodeling, Peking University, Beijing, 100191 China; 2 Department of Anesthesiology, Peking University Third Hospital, Peking University, Beijing, 100091 China; 3 Postdoctoral Station of Basic Medicine, the Second Xiangya Hospital, Central South University, Changsha, 410000 China; 4 Postdoctoral Station of Basic Medicine, the Third Xiangya Hospital, Central South University, Changsha, 410000 China; 5 Department of Anesthesiology, Longhua Hospital, Shanghai University of Traditional Chinese Medicine, Shanghai, 200032 China

**Keywords:** Acute lung injury, Gαq/11, Neutrophil extracellular traps, ER stress, IRE1α

## Abstract

**Background:**

Acute lung injury (ALI) is distinguished by exaggerated neutrophil extracellular traps (NETs), elevated clinical mortality rates, and a paucity of targeted therapeutic interventions. The Gαq/11 protein, a member of the G protein subfamily, is an effective intervention target for a variety of diseases, but little is known about its role in ALI.

**Methods:**

In this study, a murine model of ALI induced by lipopolysaccharide (LPS) was utilized, employing myeloid cell-specific *Gna11* knockout mice. The pulmonary pathology of mice was assessed and the lung samples were collected for immunofluorescence staining and RNA-sequencing analysis to elucidate the impact and underlying mechanisms of Gαq/11 in ALI. Mouse bone marrow-derived neutrophils were isolated and cultured for live-cell imaging to investigate the in vitro effects of Gαq/11.

**Results:**

The expression of Gαq/11 was found to be upregulated in the lung tissues of mice with ALI, coinciding with the increased expression of inflammatory genes. Myeloid cell-specific *Gna11* deficience attenuated LPS-induced lung injury and the formation of NETs in mice. Mechanistically, Gαq/11 facilitates NETosis by promoting the activation of the endoplasmic reticulum (ER) stress sensor IRE1α in neutrophils and mediating the production of mitochondrial reactive oxygen species (mitoROS). Pharmacological inhibition of Gαq/11 using YM-254,890 was shown to reduce NETs formation and lung injury in mice.

**Conclusions:**

The upregulation of Gαq/11 exacerbates ALI through the promotion of ER stress-mediated NETosis. Consequently, Gαq/11 represents a potential therapeutic target for the treatment of ALI.

**Supplementary Information:**

The online version contains supplementary material available at 10.1186/s10020-025-01118-4.

## Introduction

Acute lung injury (ALI) is an abrupt respiratory disorder characterized by noncardiogenic bilateral pulmonary edema and hypoxemia, attributable to damage to the pulmonary endothelial barrier and increased alveolar capillary permeability, with in-hospital mortality rates ranging from 38 to 46% (Meyer et al. 2021). The primary management strategies are symptomatic and supportive, encompassing ventilatory support, prone positioning, fluid management, and the administration of glucocorticoids (Laffey and Kavanagh 2017). Despite decades of extensive research into pharmacological interventions for ALI, no effective therapeutic agents have been identified (Butt et al. 2016). Consequently, there is an urgent need to further investigate the underlying mechanisms of ALI and to identify novel potential therapeutic targets.

In ALI, the aggregation and infiltration of neutrophils within the pulmonary tissue substantially affect the progression of the disease (Burn et al. 2021). Neutrophils modulate the inflammatory response through various mechanisms, with the release of neutrophil extracellular traps (NETs) serving as a pivotal process (Castanheira and Kubes 2019). The release of NETs predominantly takes place via a cell death mechanism known as NETosis. NETs are intricate networks of chromatin fibers released by activated neutrophils, comprising DNA, histones, and proteolytic enzymes such as myeloperoxidase (MPO) and neutrophil elastase. NETs entrap and eliminate pathogens, thereby inhibiting their dissemination (Papayannopoulos 2018). Nonetheless, excessive NETs formation can induce significant inflammation and tissue damage, thereby exacerbating the progression of ALI and elevating its associated morbidity and mortality rates (Herre et al. 2023; Xie et al. 2023). Consequently, therapies aimed at targeting NETs are regarded as a viable treatment strategy for ALI, with the potential to mitigate pulmonary symptoms (Mutua and Gershwin 2021).

In humans, chemoattractants such as chemotactic lipids, formyl peptides, complement anaphylatoxins, and chemokines exert their effects by activating specific heptahelical G protein-coupled receptors (GPCRs), including CXCR2 and CCR2, located on the surface of neutrophils(Kienle et al. 2021). This activation plays a crucial role in the precise regulation of neutrophil storage in the bone marrow and their subsequent egress, as well as in the spatial and temporal trafficking of neutrophils between different organs (Wang et al. 2023b). G proteins, which are intramembrane proteins associated with GPCRs, function as “molecular switches” that facilitate the transmission of downstream signals related to GPCR activation and are directly involved in a variety of physiological and pathophysiological processes of human body (Zhang and Shi 2016). The Gαq/11 protein, a member of the G protein subfamily, has been previously identified as an effective target for uveal melanoma treatment (Ge et al. 2022). Recent investigations have elucidated that Gαq/11 proteins play a significant role in signaling pathways that modulate inflammatory responses, which are pivotal in the context of ALI (Kamato et al. 2015; Kostenis et al. 2020). The observed correlation between chemokines such as CXCL9 and CXCL10 and acute allograft injury implies that Gαq/11 may also be implicated in the recruitment of mononuclear cells during episodes of acute rejection and ALI (Chen et al. 2019; Shino et al. 2022). The elevated concentrations of these chemokines in bronchoalveolar lavage fluid (BALF) during such events further substantiate the hypothesis that Gαq/11 signaling may play a pivotal role in the pathogenesis of ALI. However, the involvement of Gαq/11 signaling in the regulation of neutrophil function, as well as its specific role in ALI models, remains unexplored.

This study employed myeloid cell-specific *Gna11* knockout mice to develop a lipopolysaccharide (LPS)-induced ALI model, with the objectives of elucidating the specific role of Gαq/11 in ALI, uncovering its underlying mechanisms, and identifying potential novel targets for therapeutic intervention in ALI.

## Material and method

### Animals

All mice (male, 8–10 weeks old) were generated on a C57BL/6 background and the myeloid cell-specific *Gna11* knockout (*Gna11*^*fl/fl*^*Lyz2Cre*) mice were generated by crossing *Gna11*^*floxlflox*^ with *Lyz2-cre* mice. *Gna11*^*floxlflox*^*mice* (NM-CKO-205090) were purchased from Shanghai Model Organisms Center Inc. (Shanghai, China). *Lyz2-cre* mice has been described in our previous studies (Wang et al. 2023a). Mice were maintained under SPF conditions with a 12-hour light-dark cycle, and had unlimited access to food and water. Environmental conditions were controlled for humidity (60–80%) and temperature (22 ± 1 °C). All animal experiments were performed in accordance with local guidelines for the care and use of laboratory animals, as stipulated by the Laboratory Animal Ethics Committee of the Peking University.

### In vivo experiments

To establish ALI model, mice were anesthetized using sodium pentobarbital at a dosage of 50 mg/kg, then an aerosol injector was used to single orotracheal atomizing injection of LPS (Sigma, MO, USA) (10 mg/kg) suspension from the throat into the bronchus. The mice were subsequently sacrificed at 24 h. BALF and lung tissues were collected for further analysis. Cells in the BALF were collected and subsequently stained with anti-Ly6G-PE and anti-CD11b-APC (eBiosciences, CA, USA) for the identification of neutrophils utilizing flow cytometry. The methodologies for assessing the lung wet-to-dry weight ratio and protein concentration in BALF have been previously documented (Wang et al. 2023a). YM-254,890 (MCE, China) was dissolved in 2% dimethyl sulfoxide (DMSO) and administered intraperitoneally at dose of 0.15 or 0.3 mg/kg 3 h after LPS challenge.

### Quantitative real-time PCR (q-PCR)

Total mRNA was extracted using a rapid extraction kit (220010; Fastegen, Shanghai, China) according to the manufacturer’s instructions. cDNA was synthesized from 6.5 µg of mRNA using a PrimeScript RT-PCR Kit (RR014A, Takara Bio, Maebashi, Japan). mRNA levels were quantified by q-PCR using TB Green Premix Ex Tap (RR420A, Takara Bio). Gene expression was normalized to that of the housekeeping gene *B2M* according to the 2^-ΔΔCt^ method. The primers used in this study are listed in Table 1.

**Table 1:** PCR primer sequences

Gene	Forward primer (5′-3′)	Reverse primer (5′-3′)
*Gna11*	CAACGCGGAGATCGAGAAACA	CAGTGCCAAGTAGCAGCAG
*TNF-α*	AATGGCCTCCCTCTCATCAG	CCCTTGAAGAGAACCTGGGA
*IL-6*	TACCACTCCCAACAGACCTG	GGTACTCCAGAAGACCAGAGG
*IL-1β*	CTCACAAGCAGAGCAAGC	CAGTCCAGCCCATACTTAG
*PDI*	CAAGATCAAGCCCCACCTGAT	AGTTCGCCCCAACCAGTACTT
*BiP*	CAGCCAATTATCAGCAAACTCT	CAACTCCACTCTGAGGTGAAG
*PERK*	TAGGAAGATTCGAGCAGGGA	CTAGCCTCAGCAAGCCAGAG
*ATF6*	GAAGACTGGGAGTCGACGTT	ACTCCCAAGGCATCAAATCCAA
*ATF4*	CCTTCGACCAGTCGGGTTTG	CTGTCCCGGAAAAGGCATCC
*CHOP*	CGGAACCTGAGGAGAGAGTG	GTCTCCAAGGTGAAAGGCAG
*sXBP1*	GGTCTGCTGAGTCCGCAGCAGG	GGGGAAGGACATTTGAAACA
*tXBP1*	CTGAGCCCGGAGGAGAAA	TGCTCCAGCTCGCTCATC
*B2M*	CGGCCTGTATGCTATCCAGA	GGGTGAATTCAGTGTGAGCC

### Enzyme-linked immunosorbent assays (ELISA)

Following the manufacturer’s instructions, ELISA kits (Invitrogen, Carlsbad, CA, USA) were used to detect cytokines (TNF-α, IL-6, and IL-1β) in BALF and cell culture supernatant. The matrix metalloproteinase-9 (MMP-9) activity in BALF were determined by Mouse MMP-9 PicoKine™ ELISA Kit (Boster Biological, CA, USA). The peroxidase (POD) activity in BALF was measured using POD thekit (Nanjing jiancheng Bioengineering Institute, Nanjing, China). Briefly, 150 µL TMB substrate solution was added to 50 µL of BALF and incubated at room temperature for 30 min prior to termination with 50 µL 1M H_2_SO_4_. Plates were read spectrophotometrically at 450 nm. Absorbance values for POD activity were divided into the average of values from 0.5% CHAPS-lysed cells to give percentage of total cellular mediator released.

### Detection of MPO-DNA complexes

The MPO antibody (5 µg/mL, Invitrogen, CA, USA) was applied to a streptavidin-coated plate from the Cell Death Detection ELISA kit (Roche, Basle, Switzerland) and incubated at 4 °C overnight. Subsequently, the plates were washed three times with PBST (0.05% Tween-20 in PBS) and blocked with 1% BSA in PBS for 1 h at room temperature (RT). For detection, 100 µL of samples were added to each well and incubated for 2 h at RT with shaking. Following three washes, 100 µL of anti-DNA-POD from the Cell Death Detection ELISA kit (Roche) was added to each well and incubated for 2 h at RT with shaking. Subsequently, after an additional three washes, 100 µL of peroxidase substrate (ABTS, Roche, IN, USA) was added. Absorbance was measured at a wavelength of 405 nm after a 20-minute incubation period at RT in the dark.

### Hematoxylin and eosin (H&E), and immunofluorescence (IF) staining of lungs

Lung tissue samples were fixed in 4% paraformaldehyde for 24 h, subsequently embedded in paraffin, and sectioned. Following deparaffinization, sections were stained with H&E. Alveolar congestion, hemorrhaging, inflammatory cell aggregation, and alveolar wall thickness were evaluated using light microscopy. Lung injury scores, ranging from 0 to 5, were assigned according to established criteria (Matute-Bello et al. 2011). For IF analysis, sections were incubated with primary Gαq/11 (1:1000, sc-515689) (Santa Cruz Biotechnology, TX, USA), MPO (1:1000, PA5-16672) (Invitrogen, NY, USA) and Citrullinated histone H3 (Cit H3, 1:1000, ab5103) (Abcam, Cambridge, UK) antibodies at 4 °C overnight following a blocking step. Secondary antibodies conjugated with different fluorophores were incubated with tissue sections for 1 h at RT. Following rinsing and mounting with glycerol, images were captured utilizing a fluorescence microscope.

### Total NETs score in lung tissues

The quantification and scoring of NETs were conducted in accordance with the methodology outlined in a previous study (Moorthy et al. 2016). In summary, DAPI, MPO, and Cit H3 were co-stained to distinguish the two primary morphological patterns of NETs formation: individual single strands and clusters of dsDNA. The areas of these clusters, measured using ImageJ, served as the scoring criteria. The total score was derived by summing the scores from 20 distinct fields.

### RNA sequencing and bioinformatics

RNA isolation was conducted utilizing the TRIzol reagent (Invitrogen). Following isolation, RNA-seq libraries were prepared using the VAHTS Universal V6 RNA-seq Library Prep Kit for Illumina (Vazyme, Nanjing, China) in accordance with the manufacturer’s protocol. Sequencing was executed on a DNBSEQ-T7 sequencer (BGI). The untrimmed raw reads were pseudo-aligned to the Mus musculus transcriptome (Ensembl v79) and quantified at the transcript level using Salmon v1.8.0. (Patro et al. 2017). The count matrix was subsequently generated utilizing the tximport package (v1.32.0), and differential gene expression analysis was conducted employing the DESeq2 package (v1.44.0) (Soneson et al. 2015). Pathway enrichment analysis was carried out using the clusterProfiler package (v4.12.0) (Wu et al. 2021). To augment data visualization, the R packages ggGenshin (v0.1.0) and enrichplot (v1.24.0) were employed.

### Isolation and stimulation of murine neutrophils

Mouse neutrophils were isolated from bone marrow using a modified Percoll gradient centrifugation method. Bone marrow cells were collected in ice-cold HBSS (without Ca2^+^ and Mg^2+^, with 0.5% BSA), centrifuged at 500 × g for 5 min at 4 °C, then layered on a 3-step Percoll gradient (78%, 65%, 55%) and centrifuged at 800 × g for 20 min at RT. Cells at the 78%-65% interface were collected and washed. Neutrophil viability post-purification was over 95%, confirmed by trypan blue staining. Purity exceeded 90%, confirmed by flow cytometry for CD11b^+^ and Ly6G^+^ cell surface markers.

NETs from LPS-activated neutrophils were collected as previously described (Yang et al. 2023). Briefly, freshly isolated neutrophils were plated in 6-well culture plates (4 × 10^6^ cells/mL) and stimulated with LPS (10 µg/mL) for 4 h. Neutrophils were conditioned with the IRE1α inhibitors 4µ8C (25 µM, Sigma) for 30 min prior to stimulation with LPS. After a 4-hour incubation period, the neutrophils were stained, and the supernatant was collected for ELISA. All cell cultures were maintained at 37 °C with 5% CO_2_ in a dark environment.

### Visualization of NETs in vitro

For live-cell imaging, neutrophils were cultured on confocal dishes and stained with the impermeable DNA dye Sytox Green (1 µM, Thermo-Fisher Scientific). Following NETs induction, a period of 10–20 min was allocated for configuring the imaging parameters of the Leica TCS SP8 confocal microscope. The microscope was equipped with a temperature-controlled chamber maintained at 37 °C. Sytox Green intensity was counted in three random fields per sample. For the endpoint visualization of NETs, cells were seeded onto 12-mm glass coverslips and subsequently stained with both Sytox Orange (Invitrogen), a cell-impermeable dye, and Syto 13 (Invitrogen), a cell-permeable dye. Following the induction of NETs, samples were fixed with 3% PFA. Images were acquired after a 4-hour incubation period. Neutrophils positive for Sytox Orange were identified as undergoing NETosis. The quantification of NETosis was performed by enumerating these cells across three randomly selected fields per sample.

### Detection of mitochondrial ROS production

To detect mitochondrial ROS, MitoSOX (Thermo-Fisher Scientific) was employed in accordance with the manufacturer’s protocol. A total of 5 × 10^5^ cells were incubated with 5 µM MitoSOX for 20 min. Subsequently, the cells were washed three times with serum-free RPMI 1640 medium. Quantitative analysis was performed using flow cytometry, and the data were analyzed with FlowJo software (Tree Star, Inc., Ashland, OR).

### Western blot analysis

Neutrophils were lysed in a cell lysis buffer composed of 50 mM Tris-HCl (pH 8.0), 2% SDS, and 10% glycine, supplemented with protease inhibitor cocktails. Protein concentrations were quantified using a BCA protein assay kit. Equal amounts of total protein (20–30 µg per sample) were resolved on a 10–15% SDS-PAGE gel and subsequently transferred onto a PVDF membrane via electrophoresis. The membranes were blocked and incubated with primary antibodies against Gαq/11 (1:2000, sc-515689) (Santa Cruz Biotechnology, TX, USA), p-IRE1α (1:2000, NB100-2323) (Novus biologicals, Littleton, USA), IRE1α (1:2000, 3294 S) (Cell Signaling Technology, Boston, USA), s-XBP1 (1:2000, 12782 S) (Cell Signaling Technology, Boston, USA), t-XBP1 (1:2000, ab37152) (Abcam, Cambridge, UK) and β-actin antibody (1:2000, 3700 S) (Cell Signaling Technology) at 4 °C overnight. Following three washes with TBST, the membranes were incubated with specific secondary antibodies for 2 h at RT. The signals were then imaged with the ImageQuant LAS4000 mini system (GE healthcare).

### Statistical analysis

Statistical analysis was performed using GraphPad Prism software (version 9.0; GraphPad Software, CA, USA). Data were analyzed using two-tailed Student’s *t* test or one-way analysis of variance (ANOVA) followed by Dunnett’s post hoc *t* tests. Data are presented as the mean ± standard error of mean (SEM). Statistical significance was set at *P* < 0.05.

## Results

### Gαq/11 is up-regulated in the lung tissues of ALI mice

**Fig. 1:**
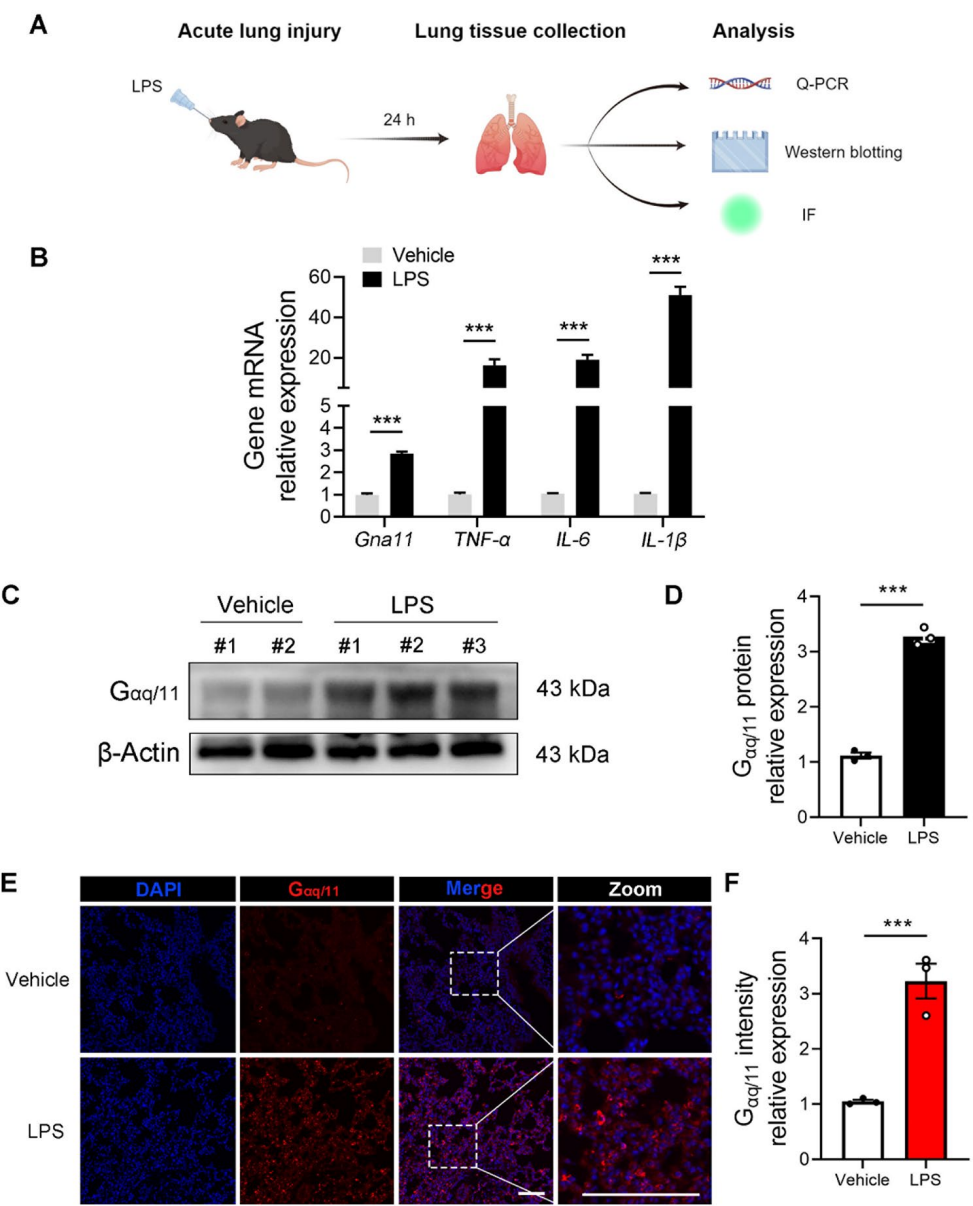
Gαq/11 is up-regulated in the lung tissues of ALI mice. **(A)** Experimental scheme for determining mRNA and protein expression of Gαq/11 in the ALI model. **(B)** mRNA relative expression of *Gna11*, *TNF-α*, *IL-6* and *IL-1β* was measured using q-PCR. **(C**,** D)** The expression level of Gαq/11 was determined through western blotting, with its relative expression depicted in bar graphs. (*n* = 3 independent biological repeats, # stands for a mouse) **(E**,**F)** Representative immunofluorescence staining and quantification of Gαq/11 (in red) (scale bar: 200 μm). The results shown were representative of three independent experiments. Data are expressed as the mean ± SEM. *P < 0.05; **P < 0.01; ***P < 0.001 as indicated

To elucidate the role of Gαq/11 in the development of ALI, we developed a model of orotracheal LPS-induced ALI to examine the expression of Gαq/11 in lung tissues (Fig. 1A). The timing and dosage of LPS administration were selected to replicate the onset of the acute phase of lung injury. Our findings indicated that, concurrent with the upregulation of genes associated with inflammatory mediators such as TNF-α, IL-6, and IL-1β, there was a marked increase in the mRNA expression of Gαq/11 in the lungs of mice with ALI (Fig. 1B). Moreover, our study revealed a marked upregulation of Gαq/11 protein levels in the lung tissues of mice with ALI (Fig. 1C, D). IF analyses further corroborated the increased expression of Gαq/11 in the lungs of ALI-affected mice (Fig. 1E, F). Taken together, these findings suggest a potential involvement of Gαq/11 in the Inflammatory cascade of ALI.

### *Gna11* deficiency alleviates ALI in mice

**Fig. 2:**
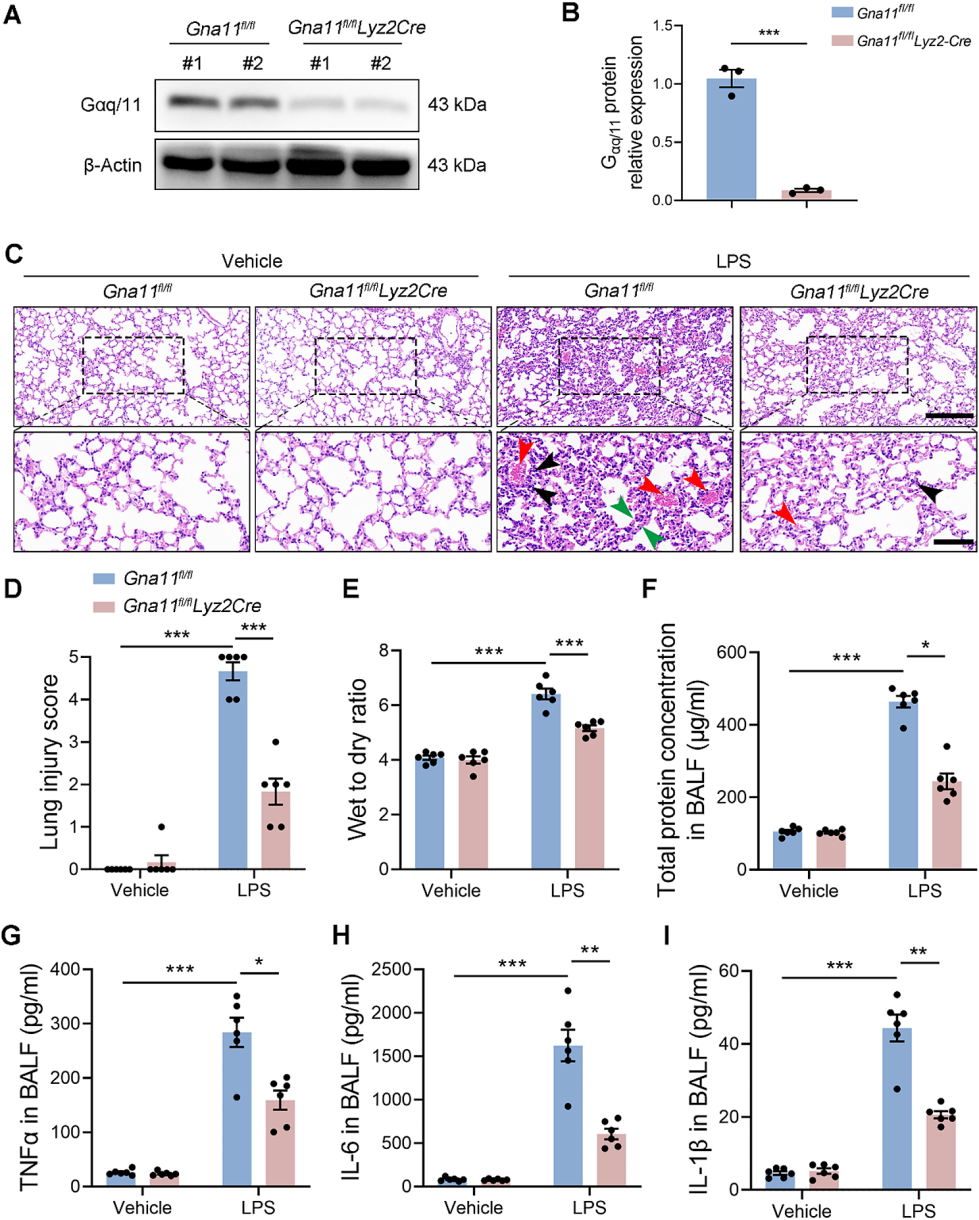
*Gna11* deficiency alleviates ALI in mice. **(A**,** B)** The expression levels of Gαq/11 protein in cells present in BALF were determined by western blotting and relative expression of Gαq/11 were shown as bar graphs. (*n* = 3 independent biological repeats, # stands for a mouse) **(C)** Representative H&E staining of the lungs (scale bars, 200 μm, 50 μm. Black arrow, inflammatory cell infiltration. Green arrow, alveolar walls thickness. Red arrow, congestion and hemorrhage). **(D)** Lung injury score of sections. **(E)** Lung wet to dry ratio. **(F)** Total protein concentrations in BALF. The concentrations of TNF-α**(G)**, IL-6**(H)** and IL-1β**(I)** in BALF were measured. The results shown were representative of six independent experiments. Data are expressed as the mean ± SEM. *P < 0.05; **P < 0.01; ***P < 0.001 as indicated

To examine the functional significance of immune cell Gαq/11 in the context of ALI, we generated myeloid cell-specific *Gna11* knockout (*Gna11*^*fl/fl*^*Lyz2Cre*) mice. The successful knockout of Gαq/11 at the protein level of immune cell in BALF was confirmed via western blot analysis (Fig. 2A, B). No metabolic or hemostatic disturbances or significant changes in blood cell counts and blood pressure were observed in *Gna11*^*fl/fl*^*Lyz2Cre* mice (Fig. S1A-F). We conducted a comparative analysis of lung tissue histopathology between *Gna11*^*fl/fl*^ littermates and *Gna11*^*fl/fl*^*Lyz2Cre* mice following the induction of ALI. H&E staining demonstrated a reduction in alveolar wall thickness, inflammatory cell infiltration, and alveolar collapse in the lungs of LPS-injured *Gna11*^*fl/fl*^*Lyz2Cre* mice compared to *Gna11*^*fl/fl*^ littermates (Fig. 2C, D). Furthermore, *Gna11*^*fl/fl*^*Lyz2Cre* mice exhibited a reduction in pulmonary injury and edema, as evidenced by a decreased lung wet-to-dry ratio and lower protein concentrations in BALF (Fig. 2E, F). The pathogenesis of ALI is significantly influenced by exaggerated host immune responses; thus, effective management of the intrapulmonary inflammatory response can either decelerate the progression of ALI or enhance survival rates. Notably, a reduction in TNF-α, IL-6, and IL-1β levels in the BALF of LPS-challenged mice was observed to be contingent upon *Gna11* deficiency (Fig. 2G-I). Collectively, our findings indicate that myeloid cell-specific *Gna11* deficiency mitigates lung damage and inflammatory responses in ALI.

### Gαq/11 contributes to NETs formation in ALI mice

Given the pivotal role of macrophages and neutrophils in the inflammatory response associated with ALI (Schulz et al. 2021), this study examined the influence of Gαq/11 on these specific inflammatory cell types. Our findings demonstrated that *Gna11* deficiency did not affect macrophage infiltration in mouse lungs or alter TNF-α and IL-6 levels in LPS-stimulated bone marrow-derived macrophages (Fig. S1B-D), suggesting Gαq/11 does not influence macrophage function in ALI.

**Fig. 3:**
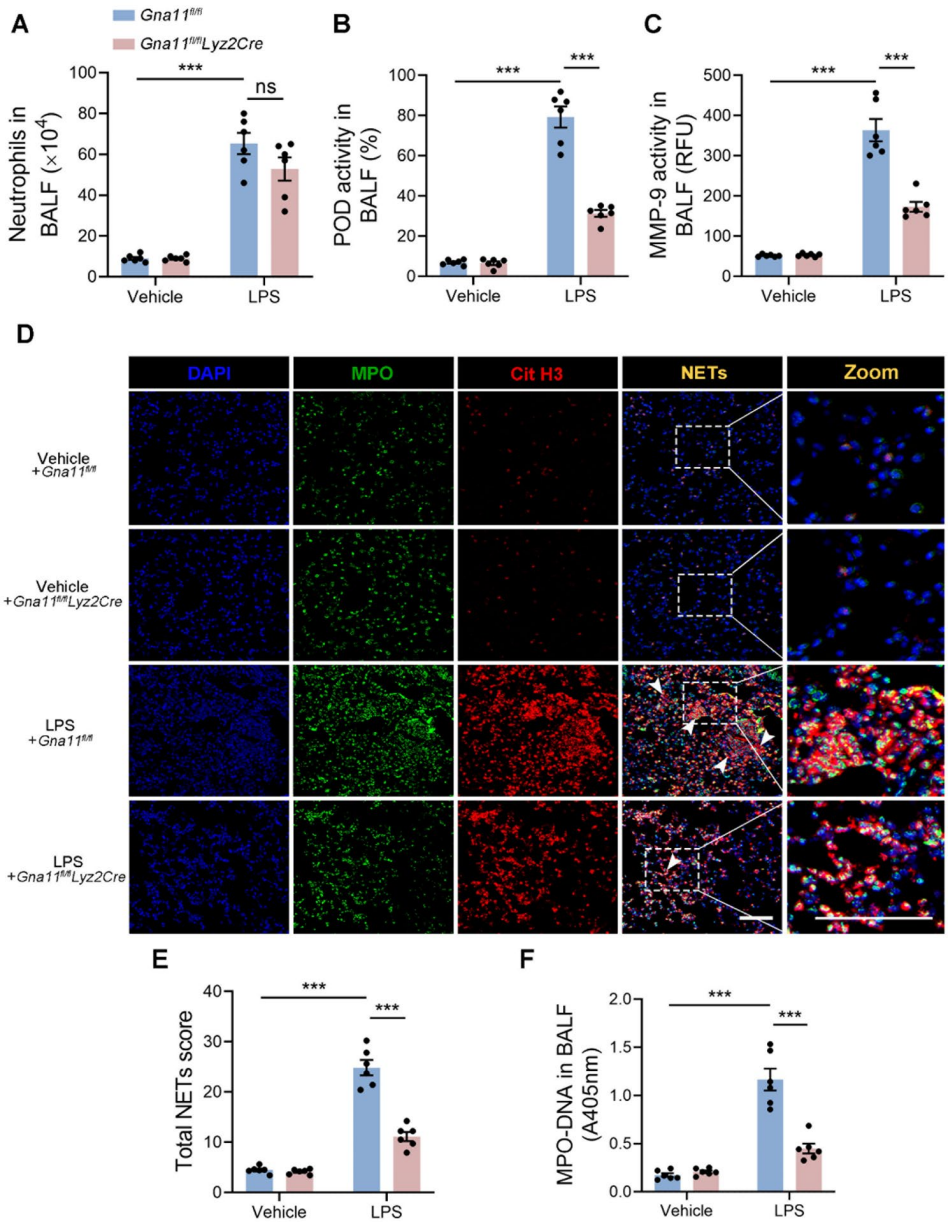
Gαq/11 contributes to NETs formation in ALI mice. **(A)** The numbers of neutrophils in BALF. **(B)** POD activity in BALF. **(C)** MMP-9 activity in BALF. **(D)** Representative immunofluorescence staining of NETs formation in the lung tissues of mice (DAPI: blue; MPO: green; Cit H3: red; Merge: Cit H3 ^**+**^ MPO ^**+**^ NETs; scale bars, 100 μm). **(E)** Total NETs score of respective lung section was calculated. **(F)** MPO-DNA complexes in BALF. The results shown were representative of six independent experiments. Data are expressed as the mean ± SEM. *P < 0.05; **P < 0.01; ***P < 0.001 as indicated

Subsequently, we examined the influence of Gαq/11 on pulmonary neutrophil infiltration in the context of ALI. Our results demonstrated a trend towards a reduction in the number of infiltrated neutrophils in the lungs of *Gna11*^*fl/fl*^*Lyz2Cre* mice compared to *Gna11*^*fl/fl*^ littermates; however, this difference did not reach statistical significance (Fig. 3A). Furthermore, myeloid cell-specific *Gna11* deficiency was observed to attenuate the increase in LPS-induced neutrophil activation markers, such as POD and MMP-9 (Fig. 3B, C). Given the pivotal role of NETs in neutrophil-mediated inflammation, we investigated the formation of NETs in *Gna11*^*fl/fl*^ and *Gna11*^*fl/fl*^*Lyz2Cre* mice. Subsequently, the expression levels of MPO (a classic neutrophil marker) and Cit H3 (a marker of NETosis primarily derived from neutrophils) in lung tissue were assessed using IF to evaluate NETs formation (Fig. 3D). Statistical analyses indicated that the absence of *Gna11* significantly reduced the score of LPS-induced NETs compared to *Gna11*^*fl/fl*^ littermates (Fig. 3E). Furthermore, analysis of the MPO-DNA complex in BALF demonstrated that myeloid cell-specific *Gna11* deficiency effectively decreased LPS-induced NETs levels in BALF (Fig. 3F). These findings indicate that Gαq/11 plays a contributory role in the formation of NETs in LPS-induced ALI.

### Gαq/11 facilitates NETosis in vitro

**Fig. 4:**
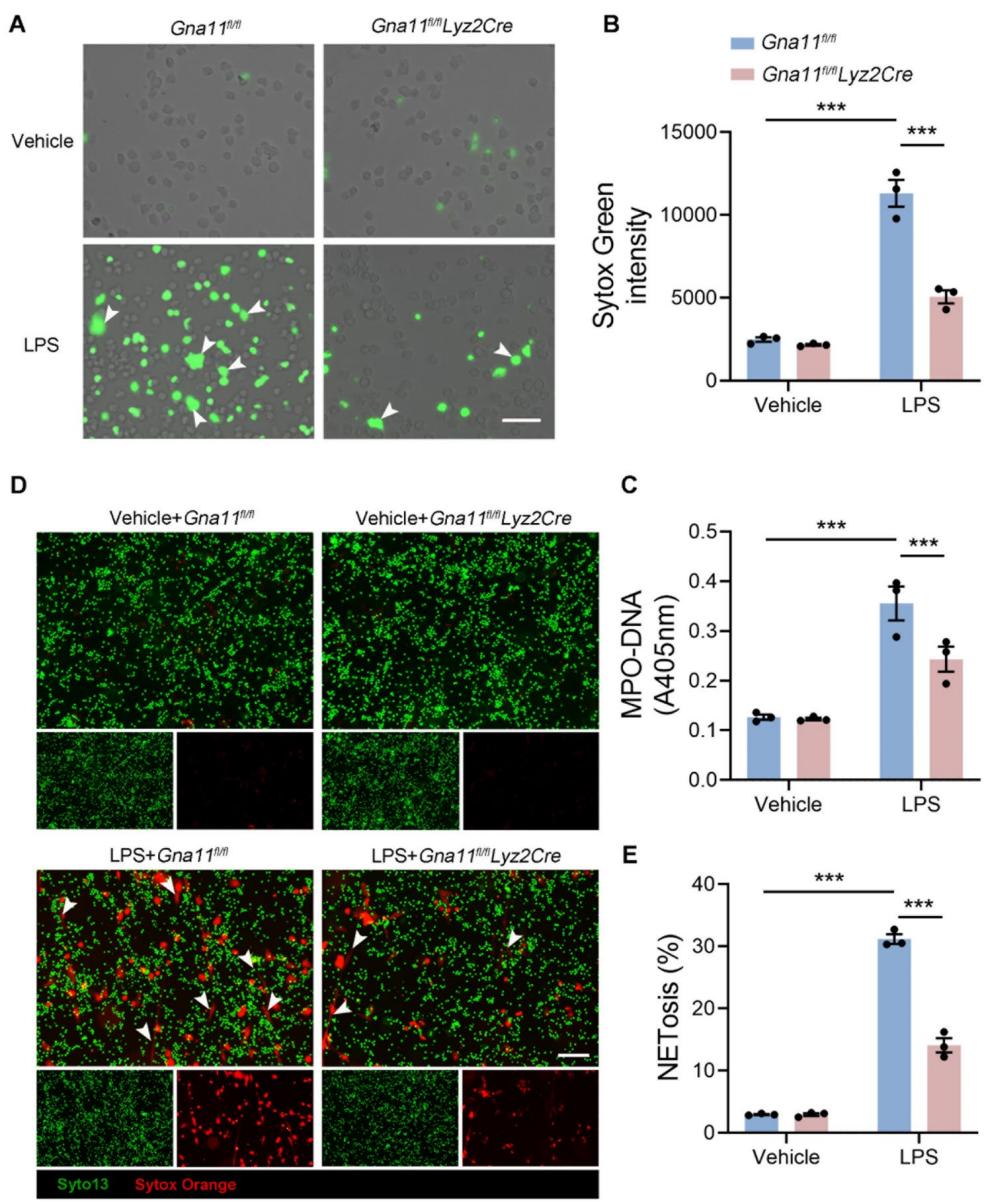
Gαq/11 facilitates NETosis *in vitro.***(A-E)** BMNs were stimulated by 10 µg/mL LPS for 4 h. **(A)** Representative fluorescence images of extracellular DNA (Sytox Green) in BMNs (scale bars, 50 μm. White arrow, Sytox Green^+^ neutrophils). **(B)** Quantitative analysis of Sytox Green intensity in supernatant. **(C)** MPO-DNA complexes in supernatant. **(D-E)** Representative immunofluorescence images of NETs formation (Syto 13 and Sytox Orange. scale bars, 200 μm. White arrow, Sytox Orange^+^ neutrophils) and quantitative analysis. The results shown were representative of three independent experiments. Data are expressed as the mean ± SEM. *P < 0.05; **P < 0.01; ***P < 0.001 as indicated

To further evaluate the influence of Gαq/11 on NETs formation, bone marrow neutrophils (BMNs) from *Gna11*^*fl/fl*^ and *Gna11*^*fl/fl*^*Lyz2Cre* mice were isolated and exposed to LPS. Extracellular DNA was visualized using Sytox Green (SG) staining, and NETs formation was quantified by assessing SG fluorescence intensity and MPO-DNA complexes in the cell supernatant. Our findings indicated that LPS stimulation induced the release of spiky NETs in WT BMNs, whereas a significant reduction in NETs formation was observed in *Gna11*^*fl/fl*^*Lyz2Cre* BMNs (Fig. 4A-C). Additionally, staining of BMNs with Syto 13 and Sytox Orange further substantiated these findings, revealing a marked decrease in LPS-induced NETosis in *Gna11*^*fl/fl*^*Lyz2Cre* BMNs (Fig. 4D, E). Collectively, our results indicate that Gαq/11 promotes the process of NETosis in vitro.

### *Gna11* deficiency down-regulates ER stress-related pathways

**Fig. 5:**
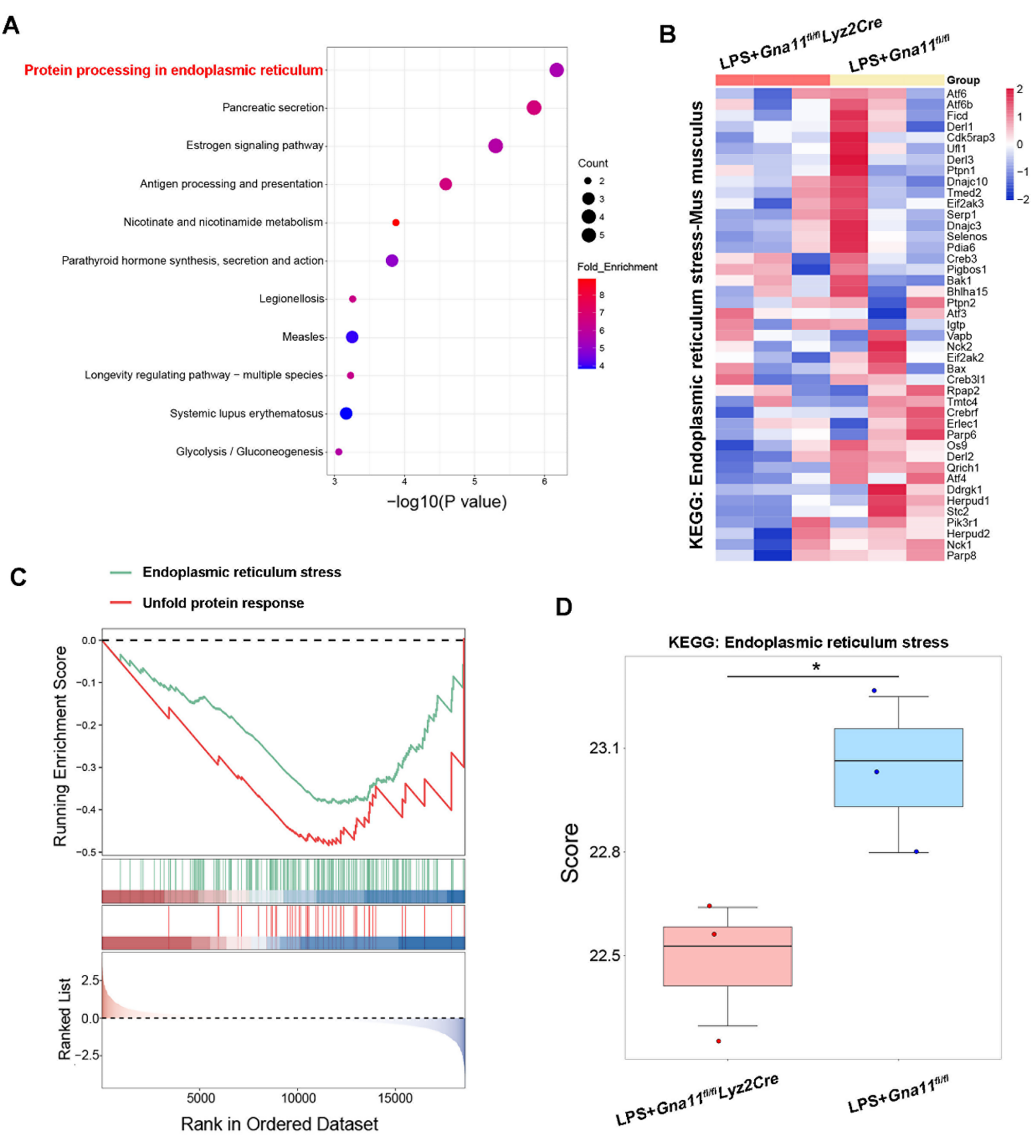
*Gna11* deficiency down-regulates ER stress-related pathways. **(A-D)** BMNs from *Gna11*^*fl/fl*^ and *Gna11*^*fl/fl*^*Lyz2Cre* mice were stimulated by 10 µg/mL LPS for 4 h and subjected to RNA sequencing (*n* = 3). **(A)** Gene ontology analysis for genes within each cluster. Bar graphs represent the top 11 enriched GO terms enriched within each cluster from Cas indicated by color. **(B)** Heat maps for differentially regulated genes involved with ER stress defined by KEGG pathway analysis. **(C)** REACTOME analysis of differentially expressed genes associated with ER stress and UPR. **(D)** Differentially activated ER stress pathway between two groups

To further elucidate the molecular mechanisms by which Gαq/11 influences the formation of NETs, BMNs isolated from *Gna11*^*fl/fl*^ and *Gna11*^*fl/fl*^*Lyz2Cre* mice treated with LPS were subjected to RNA sequencing analysis. Gene ontology enrichment analysis revealed that downregulated genes in *Gna11*^*fl/fl*^*Lyz2Cre* BMNs were predominantly associated with biological processes related to protein processing in endoplasmic reticulum (ER) (Fig. 5A). The ER, an organelle present in eukaryotic cells, is integral to numerous cellular functions, such as protein folding, calcium homeostasis, lipid metabolism, and the cellular stress response (Wang and Kaufman 2016). ER stress arises when there is a disparity between the demand for protein folding and the capacity of the ER’s folding machinery, resulting in the accumulation of misfolded proteins within the ER lumen (Walter and Ron 2011). Severe ER stress can exceed the adaptive capacity of the unfolded protein response (UPR), thereby activating apoptotic pathways and contributing to cellular dysfunction and the pathogenesis of various diseases, including ischemia/reperfusion-induced ALI (Hetz et al. 2020; Hu et al. 2015). Recent research has elucidated the potential involvement of neutrophil ER stress in the process of NETosis (Yang et al. 2024). Importantly, subsequent analysis of RNA sequencing data indicated an enrichment of biological pathways related to ER stress and UPR in *Gna11*^*fl/fl*^ BMNs infected with LPS compared to *Gna11*^*fl/fl*^*Lyz2Cre* BMNs, as evidenced by Kyoto Encyclopedia of Genes and Genomes (KEGG) and REACTOME Canonical Pathway analyses (Fig. 5B-D). These findings suggest that Gαq/11 may modulate the formation of NETs by affecting pathways associated with ER stress.

### Gαq/11 promotes the activation of ER stress sensor IRE1α

**Fig. 6:**
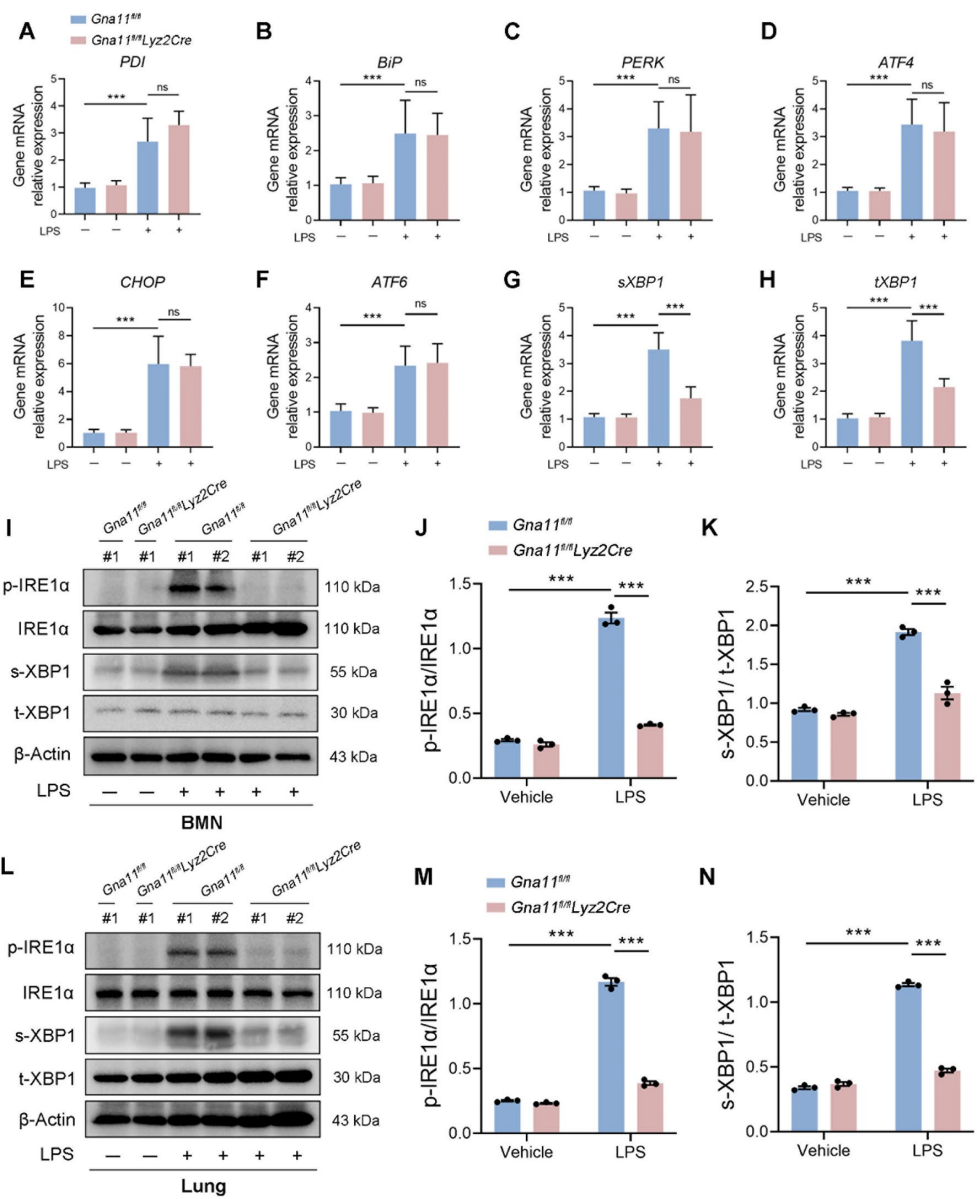
Gαq/11 promotes the activation of ER stress sensor IRE1α. **(A-H)** BMNs (2 × 10^6^ in each group, collected from 2 ~ 3 mice) were stimulated with or without 10 µg/mL LPS for 4 h, and subsequent total RNA was isolated for q-PCR analysis. mRNA levels of ER stress markers in BMNs. **(I-K)** BMNs (5 × 10^6^ in each group, collected from 6 ~ 8 mice) were treated with or without 10 µg/mL LPS for 4 h and harvested for further analysis. **(I)** The expression of p-IRE1α, IRE1α, s-XBP1 and t-XBPA protein in BMNs were detected by western blotting. **(J**,**K)** Relative expression of p-IRE1α/IRE1α and s-XBP1/t-XBPA level were shown as bar graphs. **(L)** The expression of p-IRE1α, IRE1α, s-XBP1 and t-XBPA protein in lung tissues of mice were detected by western blotting. **(M**,** N)** Relative expression of p-IRE1α/IRE1α and s-XBP1/t-XBPA level were shown as bar graphs. The results shown were representative of three independent experiments and # stands for a mouse. Data are expressed as the mean ± SEM. *P < 0.05; **P < 0.01; ***P < 0.001 as indicated

To investigate whether Gαq/11 influences ER stress in neutrophils, we examined the mRNA levels of ER stress markers, including *PDI*,* BiP*,* PERK*,* ATF4*,* CHOP*,* ATF6*,* s-XBP1*, and *t-XBP1*, in LPS-induced BMNs from *Gna11*^*fl/fl*^ and *Gna11*^*fl/fl*^*Lyz2Cre* mice. The results demonstrated that LPS stimulation significantly activated the three principal branches of ER stress in *Gna11*^*fl/fl*^ BMNs. Conversely, *Gna11* deficiency led to a marked downregulation of *s-XBP1* and *t-XBP1* (Fig. 6A-H), suggesting that Gαq/11 modulates the XBP1 splicing pathway.

IRE1α, a type 1 ER transmembrane protein kinase, undergoes oligomerization and autophosphorylation to activate its ribonuclease function in response to ER stress (Liu et al. 2017; Steiger et al. 2018). In metazoans, IRE1α catalyzes the excision of a 26-nucleotide intron from the mRNA encoding the transcription factor XBP1, resulting in a shift in the translational open reading frame. This splicing event facilitates the production of the active XBP1 transcription factor (spliced XBP1, s-XBP1), which subsequently upregulates the expression of genes associated with ER protein translocation, folding, secretion, and the degradation of misfolded proteins (Bashir et al. 2021). Furthermore, we performed an analysis to assess the influence of Gαq/11 on the activation of the IRE1α-XBP1 pathway using western blotting. The results demonstrated that the absence of *Gna11* resulted in decreased levels of LPS-induced p-IRE1α and sXBP1 (Fig. 6I-K). Additionally, our findings revealed that myeloid-specific knockout of *Gna11* led to a reduction in LPS-induced p-IRE1α and sXBP1 levels in vivo (Fig. 6L-N). Furthermore, treatment with the IRE1α inhibitor KIRA6 was shown to attenuate NETs formation in lung tissues and alleviate the severity of ALI (Fig. S2E-I). Overall, our findings suggest that Gαq/11 promotes ER stress by enhancing IRE1α activation.

### Gαq/11 accelerates mitoROS generation and NETosis through IRE1α activation

**Fig. 7:**
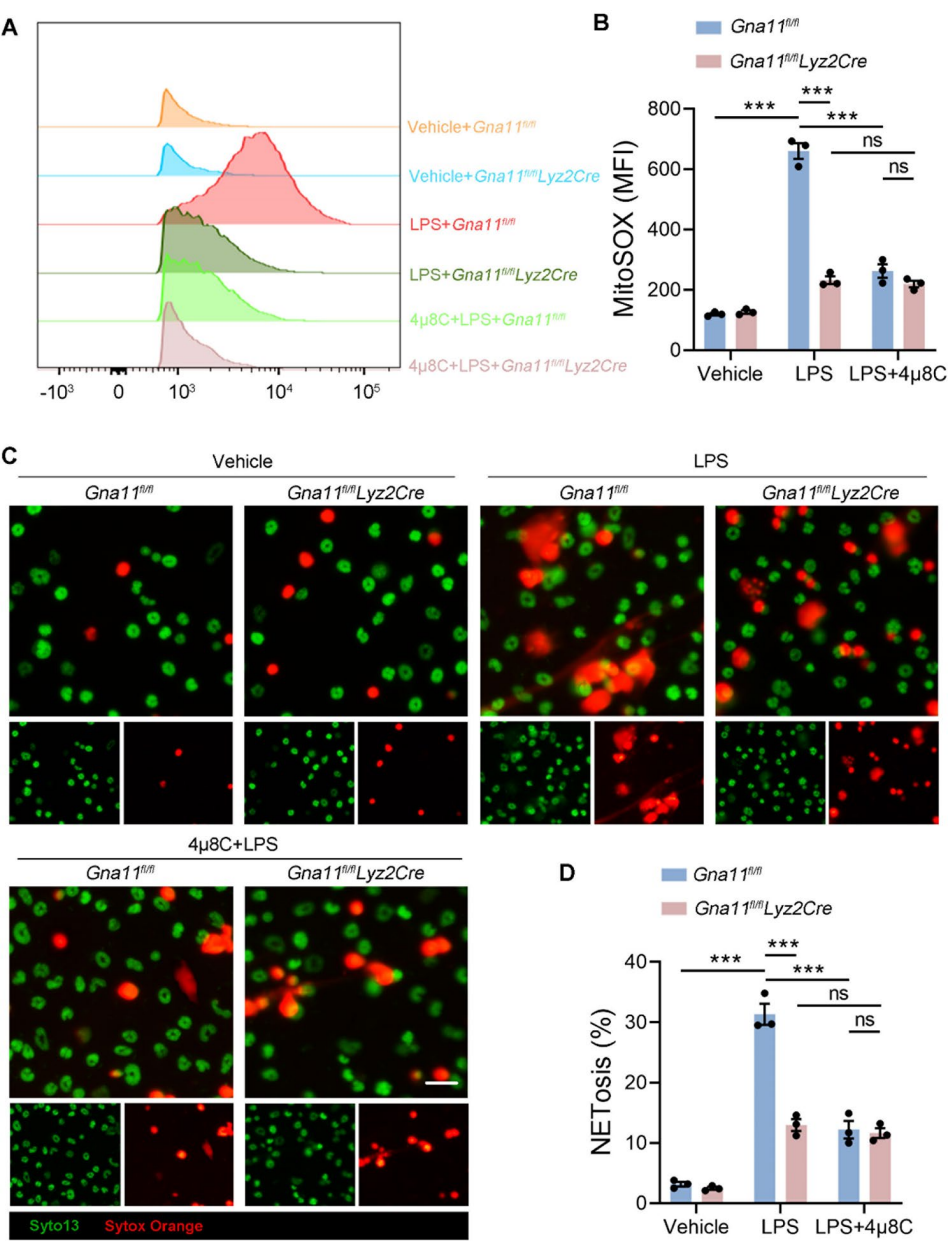
Gαq/11 accelerates mitoROS generation and NETosis through IRE1α activation. **(A**,** B)** BMNs were stained in situ using MitoSOX and signal was quantified by flow cytometry. **(C**,** D)** Representative immunofluorescence images of NETs formation (Syto 13 and Sytox Orange, scale bars, 50 μm) and quantitative analysis. The results shown were representative of three independent experiments. Data are expressed as the mean ± SEM. *P < 0.05; **P < 0.01; ***P < 0.001 as indicated

Recent research has demonstrated that the phosphorylation of IRE1α facilitates the generation of mitochondrial reactive oxygen species (mitoROS), which in turn enhances neutrophil hyperactivity and subsequent NETosis in lupus neutrophils (Sule et al. 2021). To further investigate the specific mechanisms by which Gαq/11 regulates NETosis in ALI, we conducted experiments involving the stimulation of BMNs *of Gna11*^*fl/fl*^ and *Gna11*^*fl/fl*^*Lyz2Cre* mice with LPS. MitoROS levels were quantified using flow cytometry. Our findings indicated that, relative to the vehicle control, LPS stimulation resulted in a significant increase in mitoROS levels in *Gna11*^*fl/fl*^ BMNs, as measured by the fluorescent probe MitoSOX. On the contrary, no significant increase in mitoROS levels was observed in *Gna11*^*fl/fl*^*Lyz2Cre* BMNs. Furthermore, pretreatment of BMNs with the IRE1α inhibitor 4µ8C resulted in a significant reduction in mitoROS production in *Gna11*^*fl/fl*^ BMNs. In contrast, 4µ8C did not exert a significant effect on mitoROS production in *Gna11*^*fl/fl*^*Lyz2Cre* BMNs, suggesting that Gαq/11 facilitates mitoROS production through the activation of IRE1α (Fig. 7A, B). Additionally, NETosis, assessed by Syto 13 and Sytox Orange staining of BMNs, revealed that inhibition of IRE1α by 4µ8C mitigated NETosis in *Gna11*^*fl/fl*^ BMNs but had no impact on *Gna11*^*fl/fl*^*Lyz2Cre* BMNs following LPS stimulation (Fig. 7C, D). Collectively, these findings indicate that Gαq/11 enhances mitoROS production and NETosis via the activation of IRE1α.

### Gαq/11 inhibitor YM-254,890 ameliorates ALI in mice via NETosis inhibition

**Fig. 8:**
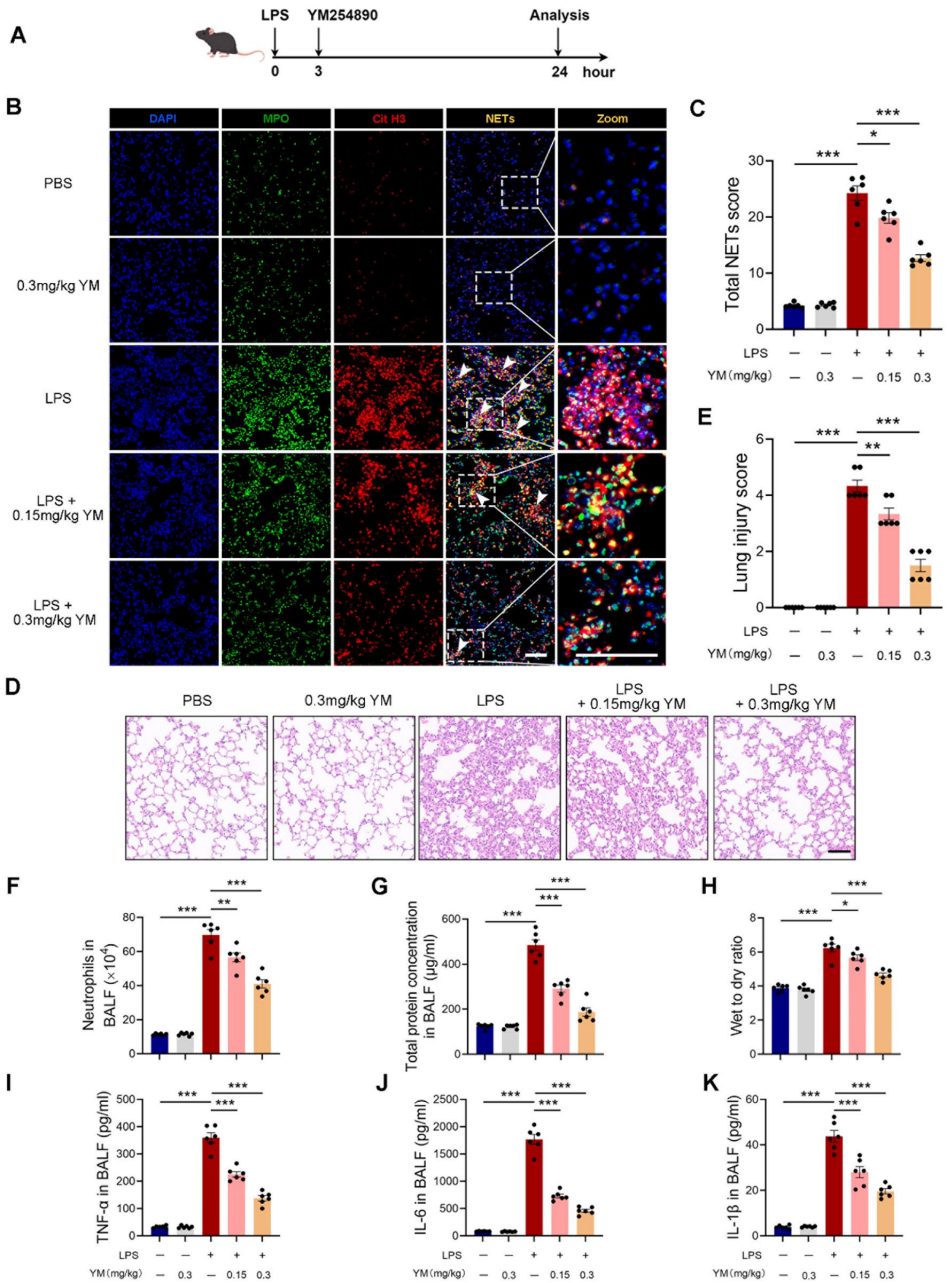
Gαq/11 inhibitor YM-254,890 ameliorates ALI in mice via NETosis inhibition. **(A)** YM254890 was administrated 3 h post-LPS challenge in mice. **(B)** Representative immunofluorescence staining of NETs in the lung tissues of mice (DAPI: blue; MPO: green; Cit H3: red; Merge: Cit H3 ^**+**^ MPO ^**+**^ NETs; scale bars, 100 μm). **(C)** Total NETs score of respective lung section was calculated. **(D)** Representative H&E staining of the lungs (scale bars, 200 μm). **(E)** Lung injury score of sections. **(F)** The numbers of neutrophils in BALF. **(G)** Total protein concentrations in BALF. **(H)** Lung wet to dry ratio. The concentrations of TNF-α **(I)**, IL-6**(J)** and IL-1β**(K)** in BALF were measured. The results shown were representative of six independent experiments. Data are expressed as the mean ± SEM. *P < 0.05; **P < 0.01; ***P < 0.001 as indicated

The aforementioned results suggest that Gαq/11 is pivotal in the progression of ALI. Consequently, the pharmacological inhibitor of Gαq/11, YM-254,890 (Uemura et al. 2006b), was employed to investigate the therapeutic potential of targeting Gαq/11 in ALI. Considering the clinical significance of therapeutic administration in ALI, YM-254,890 was administered at 3 h after LPS challenge (Fig. 8A). As seen, no metabolic or hemostatic disturbances or significant changes in blood pressure were observed after administration of YM-254,890 (Fig. S1G-I). Notably, administration of YM-254,890 at doses of 0.15 mg/kg and 0.3 mg/kg resulted in a significant reduction in the formation of LPS-induced NETs, as evidenced by decreased expression levels of MPO and Cit H3 in murine lung tissue (Fig. 8B-C). Furthermore, our results demonstrated that YM-254,890 mitigated the lung injury score, neutrophil infiltration and the degree of pulmonary edema in LPS-induced mice in a dose-dependent manner (Fig. 8D-H). Additionally, our findings revealed that treatment with YM-254,890 significantly reduced the levels of cumulative inflammatory mediators, including TNF-α, IL-6, and IL-1β, induced by LPS (Fig. 8I-K). In summary, these results suggest that YM-254,890 holds substantial therapeutic potential for inhibiting NETs and attenuating the severity of ALI.

## Discussion

This study sought to examine the role and specific mechanisms of Gαq/11 in ALI, with the aim of identifying novel potential targets for ALI drug therapy. The results demonstrated an upregulation of Gαq/11 expression in the lung tissue of a mouse model of ALI. The ablation of Gαq/11 attenuated the severity of lung injury and edema, and reduced the formation of lung NETs in LPS-induced mice. Mechanistically, the evidence suggests that Gαq/11 promotes neutrophil NETosis by enhancing the activation of the ER stress sensor IRE1α.

The potential targeting of Gαq/11 represents a pivotal area of interest in drug discovery and therapeutic development, largely due to its involvement in various diseases, including uveal melanoma and peripheral arterial disease (Mishra et al. 2010; Piaggio et al. 2022; Uemura et al. 2006b). Moreover, the broader implications of targeting GPCRs and their associated G proteins extend beyond oncology. GPCRs are recognized as the largest class of drug targets, owing to their diverse physiological functions and significant druggability (Congreve et al. 2020). By focusing on G protein subunits rather than directly on GPCRs, researchers may achieve a more comprehensive therapeutic effect, especially in complex diseases that involve multiple receptors. This strategy enables the selective inhibition of specific G protein pathways, which may lead to biased signaling and confer therapeutic benefits across various conditions, including inflammation, asthma, and heart failure (Kostenis et al. 2020). The potential role of Gαq/11 in ALI constitutes a promising research area that intersects with a wide range of immunological and pathological processes. In the context of ALI, the regulation of immune cell populations is critically important. Our research indicates that the expression of Gαq/11 is increased concurrently with the upregulation of genes related to inflammatory mediators in lung tissues in LPS-induced ALI models, suggesting a potential role in the pathogenesis of ALI. Immunofluorescence analysis of lung tissue revealed ubiquitous expression of Gαq/11 in the lungs of mice with ALI. Given the critical role of neutrophils and macrophages in the inflammatory cascade characteristic of the ALI model, we generated myeloid-specific *Gna11* knockout mice to investigate the specific function of Gαq/11 in ALI. By conducting both in vivo and in vitro experiments, we excluded the potential influence of Gαq/11 in macrophages, enabling us to focus on elucidating the specific role of neutrophil Gαq/11 in ALI pathogenesis. Furthermore, through myeloid-specific knockout of *Gna11*, we observed that the absence of Gαq/11 led to a minor, statistically insignificant decrease in neutrophil recruitment, but a significant reduction in neutrophil activation markers in ALI, highlighting its critical role in the NETs-mediated pathophysiology of ALI.

NETs have been identified as critical contributors to the pathogenesis of ALI, particularly in contexts such as transfusion-related acute lung injury and primary graft dysfunction following lung transplantation (Bonneau et al. 2022; van der Velden et al. 2024). Notably, for detecting NETs formation in lung tissues, we stained lung sections with Cit H3, a marker of NETosis, as well as MPO, a classic neutrophil marker. During NETosis, Cit H3 could be released either passively as a result of cellular injury or actively in the process of NETosis. Consequently, not all Cit H3-positive cells are also MPO-positive. Additionally, the enzyme PAD4, which catalyzes the citrullination of histones, is predominantly found in neutrophils (Li et al. 2010). Consequently, Cit H3 is primarily derived from neutrophils. Our prior investigations have elucidated the pivotal role of neutrophil GSDMD-mediated NETosis in acute respiratory distress syndrome (ARDS) (Xie et al. 2023). In the context of sepsis, excessive formation of NETs is correlated with pulmonary dysfunction, and inhibition of the gasdermin D pathway has been demonstrated to attenuate NETs release and enhance clinical outcomes (Silva et al. 2021). These findings indicate that strategies targeting the modulation of NETs formation may offer therapeutic benefits across various manifestations of ALI. Gαq/11 is recognized as a pivotal modulator of mTORC1 signaling, which is integral to autophagy and cellular homeostasis (Cabezudo et al. 2021). This signaling pathway is crucial for neutrophil functionality, particularly concerning the formation of NETs. Our research has elucidated a novel and critical function of Gαq/11 in the process of NETosis both in vivo and in vitro, thereby proposing it as a potential therapeutic target for conditions linked to NETs. Future investigations should explore the possibility that Gαq/11 modulates NETosis through its impact on the mTORC1 pathway in ALI. ER stress is triggered by the accumulation of misfolded proteins within the ER, resulting in the activation of the UPR. This response is essential for maintaining cellular homeostasis and can significantly impact the fate of neutrophils. In the context of inflammation and infection, sustained ER stress can activate pro-inflammatory pathways, potentially enhancing the formation of NETs (Chaudhari et al. 2014). Recent research has demonstrated that ER stress mediates NADPH oxidase 2 (NOX2)-dependent NETosis in a model of myocardial reperfusion injury (Yang et al. 2024). Furthermore, research has elucidated the involvement of IRE1α in the hyperactivity of neutrophils in lupus, demonstrating that this pathway precedes mitochondrial dysfunction, mitoROS formation, and NETosis. Inhibition of the IRE1α pathway thus emerges as a novel strategy for mitigating NETosis in lupus and potentially other inflammatory conditions (Sule et al. 2021). Through RNA sequencing analysis of LPS-treated *Gna11*^*fl/fl*^ and *Gna11*^*fl/fl*^*Lyz2Cre* mouse BMNs, we identified the potential regulatory function of Gαq/11 in ER stress. Furthermore, it was demonstrated that Gαq/11 facilitates NETosis by activating the IRE1α-XBP1-mitoROS signaling pathway. These findings offer a theoretical foundation for the potential therapeutic targeting of Gαq/11 in disease models such as lupus and myocardial reperfusion injury.

It is essential to acknowledge the limitations inherent in this study. Notably, the administration of the Gαq/11 inhibitor YM254,890 was systemic, affecting the entire organism in the murine model. Beyond its established function in leukocytes, Gαq/11 has been implicated in the GPR116-mediated regulation of alveolar surfactant within alveolar type II epithelial cells (Brown et al. 2017). Moreover, Gαq/11 is involved in the activation and degranulation of platelets, which interact with neutrophils, thereby enhancing their pulmonary infiltration and facilitating the formation of NETs during both infectious and non-infectious ALI (Burkard et al. 2023; Uemura et al. 2006a). Although our findings indicated that YM254,890 treatment did not significantly impact platelet counts in mice (Fig. S1H), it is possible that the ALI induced by tracheal inhalation of LPS is predominantly localized to the lung and represents an acute model. Within the complex in vivo microenvironment, YM254,890 may concurrently influence other factors to mitigate ALI.

## Conclusion

Our study elucidates the pivotal role of Gαq/11 in the ALI model and delineates the specific mechanism by which Gαq/11 mediates NETosis through the promotion of neutrophil ER stress sensor IRE1α activation. Furthermore, the therapeutic efficacy of YM-254,890, a pharmacological inhibitor of Gαq/11, is validated in the ALI model. These findings offer a novel target for the pharmacological treatment of ALI.

## Electronic supplementary material

Below is the link to the electronic supplementary material.

Supplementary Material 1

## Data Availability

No datasets were generated or analysed during the current study.

## Abbreviations

ALI: Acute Lung Injury
NETs: Neutrophil Extracellular Traps
LPS: Lipopolysaccharide
ER: Endoplasmic Reticulum
mitoROS: mitochondrial Reactive Oxygen Species
MPO: Myeloperoxidase
GPCRs: G Protein-Coupled Receptors
BALF: Bronchoalveolar Lavage Fluid
KO: Knockout
DMSO: Dimethyl Sulfoxide
ELISA: Enzyme-Linked Immunosorbent Assays
MMP-9: Matrix Metalloproteinase-9
POD: Peroxidase
RT: Room Temperature
H&E: Hematoxylin And Eosin
IF: Immunofluorescence
CitH3: Citrullinated Histone H3
ANOVA: One-Way Analysis of Variance
WT: Wild-Type
BMNs: Bone Marrow Neutrophils
UPR: Unfolded Protein Response
GSDMD: Gasdermin D
ARDS: Acute Respiratory Distress Syndrome
NOX2: NADPH Oxidase 2

## References

[CR1] Bashir S, Banday M, Qadri O, Bashir A, Hilal N, Nida IF, Rader S, Fazili KM. The molecular mechanism and functional diversity of UPR signaling sensor IRE1. Life Sci. 2021;265:118740. 33188833 10.1016/j.lfs.2020.118740

[CR2] Bonneau S, Landry C, Bégin S, Adam D, Villeneuve L, Clavet-Lanthier M, Dasilva A, Charles E, Dumont BL, Neagoe PE et al. Correlation between Neutrophil Extracellular Traps (NETs) Expression and Primary Graft Dysfunction Following Human Lung Transplantation. *Cells* 2022;11. 10.3390/cells11213420 PMC9656095 36359815

[CR3] Brown K, Filuta A, Ludwig MG, Seuwen K, Jaros J, Vidal S, Arora K, Naren AP, Kandasamy K, Parthasarathi K et al. Epithelial Gpr116 regulates pulmonary alveolar homeostasis via Gq/11 signaling. JCI Insight 2017;2. 10.1172/jci.insight.93700 PMC5453702 28570277

[CR4] Burkard P, Schonhart C, Vögtle T, Köhler D, Tang L, Johnson D, Hemmen K, Heinze KG, Zarbock A, Hermanns HM, et al. A key role for platelet GPVI in neutrophil recruitment, migration, and NETosis in the early stages of acute lung injury. Blood. 2023;142:1463–77. 37441848 10.1182/blood.2023019940

[CR5] Burn GL, Foti A, Marsman G, Patel DF, Zychlinsky A. The Neutrophil. Immunity. 2021;54:1377–91. 34260886 10.1016/j.immuni.2021.06.006

[CR6] Butt Y, Kurdowska A, Allen TC. Acute Lung Injury: a clinical and molecular review. Arch Pathol Lab Med. 2016;140:345–50. 27028393 10.5858/arpa.2015-0519-RA

[CR7] Cabezudo S, Sanz-Flores M, Caballero A, Tasset I, Rebollo E, Diaz A, Aragay AM, Cuervo AM, Mayor F Jr., Ribas C. Gαq activation modulates autophagy by promoting mTORC1 signaling. Nat Commun. 2021;12:4540. 34315875 10.1038/s41467-021-24811-4 PMC8316552

[CR8] Castanheira FVS, Kubes P. Neutrophils and NETs in modulating acute and chronic inflammation. Blood. 2019;133:2178–85. 30898862 10.1182/blood-2018-11-844530

[CR9] Chaudhari N, Talwar P, Parimisetty A, Lefebvre dâ€™Hellencourt C, Ravanan P. A molecular web: endoplasmic reticulum stress, inflammation, and oxidative stress. Front Cell Neurosci 2014;8. 10.3389/fncel.2014.00213 PMC4114208 25120434

[CR10] Chen MC, Baskaran R, Lee NH, Hsu HH, Ho TJ, Tu CC, Lin YM, Viswanadha VP, Kuo WW, Huang CY. CXCL2/CXCR2 axis induces cancer stem cell characteristics in CPT-11-resistant LoVo colon cancer cells via Gαi-2 and Gαq/11. J Cell Physiol. 2019;234:11822–34. 30552676 10.1002/jcp.27891

[CR11] Congreve M, de Graaf C, Swain NA, Tate CG. Impact of GPCR structures on Drug Discovery. Cell. 2020;181:81–91. 32243800 10.1016/j.cell.2020.03.003

[CR12] Ge Y, Deng JJ, Zhu J, Liu L, Ouyang S, Song Z, Zhang X, Xiong XF. Discovery of small molecule Gαq/11 protein inhibitors against uveal melanoma. Acta Pharm Sin B. 2022;12:3326–40. 35967274 10.1016/j.apsb.2022.04.016 PMC9366314

[CR13] Herre M, Cedervall J, Mackman N, Olsson AK. Neutrophil extracellular traps in the pathology of cancer and other inflammatory diseases. Physiol Rev. 2023;103:277–312. 35951483 10.1152/physrev.00062.2021 PMC9576172

[CR14] Hetz C, Zhang K, Kaufman RJ. Mechanisms, regulation and functions of the unfolded protein response. Nat Rev Mol Cell Biol. 2020;21:421–38. 32457508 10.1038/s41580-020-0250-z PMC8867924

[CR15] Hu R, Chen ZF, Yan J, Li QF, Huang Y, Xu H, Zhang XP, Jiang H. Endoplasmic reticulum stress of neutrophils is required for Ischemia/Reperfusion-Induced Acute Lung Injury. J Immunol. 2015;195:4802–9. 26475925 10.4049/jimmunol.1500073 PMC4635566

[CR16] Kamato D, Thach L, Bernard R, Chan V, Zheng W, Kaur H, Brimble M, Osman N, Little PJ. Structure, function, Pharmacology, and therapeutic potential of the G Protein, Gα/q,11. Front Cardiovasc Med. 2015;2:14. 26664886 10.3389/fcvm.2015.00014 PMC4671355

[CR17] Kienle K, Glaser KM, Eickhoff S, Mihlan M, Knopper K, Reategui E, Epple MW, Gunzer M, Baumeister R, Tarrant TK et al. Neutrophils self-limit swarming to contain bacterial growth in vivo. Science 2021;372. 10.1126/science.abe7729 PMC8926156 34140358

[CR18] Kostenis E, Pfeil EM, Annala S. Heterotrimeric G(q) proteins as therapeutic targets? J Biol Chem. 2020;295:5206–15. 32122969 10.1074/jbc.REV119.007061 PMC7170519

[CR19] Laffey JG, Kavanagh BP. Fifty years of Research in ARDS. Insight into Acute Respiratory Distress Syndrome. From models to patients. Am J Respir Crit Care Med. 2017;196:18–28. 28146637 10.1164/rccm.201612-2415CI

[CR20] Li P, Li M, Lindberg MR, Kennett MJ, Xiong N, Wang Y. PAD4 is essential for antibacterial innate immunity mediated by neutrophil extracellular traps. J Exp Med. 2010;207:1853–62. 20733033 10.1084/jem.20100239 PMC2931169

[CR21] Liu J, Wang Y, Song L, Zeng L, Yi W, Liu T, Chen H, Wang M, Ju Z, Cong YS. A critical role of DDRGK1 in endoplasmic reticulum homoeostasis via regulation of IRE1α stability. Nat Commun. 2017;8:14186. 28128204 10.1038/ncomms14186 PMC5290148

[CR22] Matute-Bello G, Downey G, Moore BB, Groshong SD, Matthay MA, Slutsky AS, Kuebler WM. An official American Thoracic Society workshop report: features and measurements of experimental acute lung injury in animals. Am J Respir Cell Mol Biol. 2011;44:725–38. 21531958 10.1165/rcmb.2009-0210ST PMC7328339

[CR23] Meyer NJ, Gattinoni L, Calfee CS. Acute respiratory distress syndrome. Lancet. 2021;398:622–37. 34217425 10.1016/S0140-6736(21)00439-6 PMC8248927

[CR24] Mishra S, Ling H, Grimm M, Zhang T, Bers DM, Brown JH. Cardiac hypertrophy and heart failure development through Gq and CaM kinase II signaling. J Cardiovasc Pharmacol. 2010;56:598–603. 20531218 10.1097/FJC.0b013e3181e1d263 PMC2947575

[CR25] Moorthy AN, Rai P, Jiao H, Wang S, Tan KB, Qin L, Watanabe H, Zhang Y, Teluguakula N, Chow VT. Capsules of virulent pneumococcal serotypes enhance formation of neutrophil extracellular traps during in vivo pathogenesis of pneumonia. Oncotarget. 2016;7:19327–40. 27034012 10.18632/oncotarget.8451 PMC4991386

[CR26] Mutua V, Gershwin LJ. A review of Neutrophil Extracellular traps (NETs) in Disease: potential Anti-NETs therapeutics. Clin Rev Allergy Immunol. 2021;61:194–211. 32740860 10.1007/s12016-020-08804-7 PMC7395212

[CR27] Papayannopoulos V. Neutrophil extracellular traps in immunity and disease. Nat Rev Immunol. 2018;18:134–47. 28990587 10.1038/nri.2017.105

[CR28] Patro R, Duggal G, Love MI, Irizarry RA, Kingsford C. Salmon provides fast and bias-aware quantification of transcript expression. Nat Methods. 2017;14:417–9. 28263959 10.1038/nmeth.4197 PMC5600148

[CR29] Piaggio F, Croce M, Reggiani F, Monti P, Bernardi C, Ambrosio M, Banelli B, Dogrusöz M, Jockers R, Bordo D, et al. In uveal melanoma Gα-protein GNA11 mutations convey a shorter disease-specific survival and are more strongly associated with loss of BAP1 and chromosomal alterations than Gα-protein GNAQ mutations. Eur J Cancer. 2022;170:27–41. 35580369 10.1016/j.ejca.2022.04.013

[CR30] Schulz C, Petzold T, Ishikawa-Ankerhold H. Macrophage regulation of Granulopoiesis and Neutrophil functions. Antioxid Redox Signal. 2021;35:182–91. 33107319 10.1089/ars.2020.8203

[CR31] Shino MY, Todd JL, Neely ML, Kirchner J, Frankel CW, Snyder LD, Pavlisko EN, Fishbein GA, Schaenman JM, Mason K, et al. Plasma CXCL9 and CXCL10 at allograft injury predict chronic lung allograft dysfunction. Am J Transpl. 2022;22:2169–79. 10.1111/ajt.17108 PMC9427677 35634722

[CR32] Silva CMS, Wanderley CWS, Veras FP, Sonego F, Nascimento DC, Gonçalves AV, Martins TV, Cólon DF, Borges VF, Brauer VS, et al. Gasdermin D inhibition prevents multiple organ dysfunction during sepsis by blocking NET formation. Blood. 2021;138:2702–13. 34407544 10.1182/blood.2021011525 PMC8703366

[CR33] Soneson C, Love MI, Robinson MD. Differential analyses for RNA-seq: transcript-level estimates improve gene-level inferences. F1000Res. 2015;4:1521. 26925227 10.12688/f1000research.7563.1 PMC4712774

[CR34] Steiger D, Yokota T, Li J, Ren S, Minamisawa S, Wang Y. The serine/threonine-protein kinase/endoribonuclease IRE1α protects the heart against pressure overload-induced heart failure. J Biol Chem. 2018;293:9652–61. 29769316 10.1074/jbc.RA118.003448 PMC6016466

[CR35] Sule G, Abuaita BH, Steffes PA, Fernandes AT, Estes SK, Dobry C, Pandian D, Gudjonsson JE, Kahlenberg JM, O’Riordan MX et al. Endoplasmic reticulum stress sensor IRE1α propels neutrophil hyperactivity in lupus. J Clin Invest 2021;131. 10.1172/JCI137866 PMC8011900 33561013

[CR36] Uemura T, Kawasaki T, Taniguchi M, Moritani Y, Hayashi K, Saito T, Takasaki J, Uchida W, Miyata K. Biological properties of a specific Galpha q/11 inhibitor, YM-254890, on platelet functions and thrombus formation under high-shear stress. Br J Pharmacol. 2006a;148:61–9. 16520742 10.1038/sj.bjp.0706711 PMC1617042

[CR37] Uemura T, Takamatsu H, Kawasaki T, Taniguchi M, Yamamoto E, Tomura Y, Uchida W, Miyata K. Effect of YM-254890, a specific Gαq/11 inhibitor, on experimental peripheral arterial disease in rats. Eur J Pharmacol. 2006b;536:154–61. 16566917 10.1016/j.ejphar.2006.02.048

[CR38] van der Velden S, van Osch TLJ, Seghier A, Bentlage AEH, Mok JY, Geerdes DM, van Esch WJE, Pouw RB, Brouwer MC, Jongerius I, et al. Complement activation drives antibody-mediated transfusion-related acute lung injury via macrophage trafficking and formation of NETs. Blood. 2024;143:79–91. 37801721 10.1182/blood.2023020484

[CR39] Walter P, Ron D. The unfolded protein response: from stress pathway to homeostatic regulation. Science. 2011;334:1081–6. 22116877 10.1126/science.1209038

[CR40] Wang M, Kaufman RJ. Protein misfolding in the endoplasmic reticulum as a conduit to human disease. Nature. 2016;529:326–35. 26791723 10.1038/nature17041

[CR41] Wang T, Wang Y, Xiang Q, Lin S, Jin P, Wang J, Li N, Wang J, Bian J. Deletion of G protein-coupled receptor 116 enhances neutrophil function and aggravates lung injury in mice. Genes Dis. 2023a;10:1214–6. 37397538 10.1016/j.gendis.2022.08.017 PMC10311051

[CR42] Wang Y, Zhu C-l, Li P, Liu Q, Li H-r, Yu C-m, Deng X-m, Wang J-f. The role of G protein-coupled receptor in neutrophil dysfunction during sepsis-induced acute respiratory distress syndrome. Front Immunol 2023b;14. 10.3389/fimmu.2023.1112196 PMC9986442 36891309

[CR43] Wu T, Hu E, Xu S, Chen M, Guo P, Dai Z, Feng T, Zhou L, Tang W, Zhan L, et al. clusterProfiler 4.0: a universal enrichment tool for interpreting omics data. Innov (Camb). 2021;2:100141. 10.1016/j.xinn.2021.100141 PMC8454663 34557778

[CR44] Xie J, Zhu CL, Wan XJ, Zhao ZZ, Meng Y, Li P, Guo Y, Liu Q, Bian JJ, Deng XM, et al. GSDMD-mediated NETosis promotes the development of acute respiratory distress syndrome. Eur J Immunol. 2023;53:e2250011. 36250416 10.1002/eji.202250011

[CR46] Yang K, Wu B, Wei W, Li C, Li L, Cong Z, Xiang Q. Curdione ameliorates sepsis-induced lung injury by inhibiting platelet-mediated neutrophil extracellular trap formation. Int Immunopharmacol. 2023;118:110082. 36989889 10.1016/j.intimp.2023.110082

[CR45] Yang K, Gao R, Chen H, Hu J, Zhang P, Wei X, Shi J, Chen Y, Zhang L, Chen J, et al. Myocardial reperfusion injury exacerbation due to ALDH2 deficiency is mediated by neutrophil extracellular traps and prevented by leukotriene C4 inhibition. Eur Heart J. 2024;45:1662–80. 38666340 10.1093/eurheartj/ehae205 PMC11089336

[CR47] Zhang L, Shi G. Gq-Coupled receptors in autoimmunity. J Immunol Res. 2016;2016:1–8. 10.1155/2016/3969023 PMC4739231 26885533

