# Supplementary material for: Gαq/11 aggravates acute lung injury in mice by promoting endoplasmic reticulum stress-mediated NETosis
Source: Mol Med. 2025 Feb 19;31:67. doi: 10.1186/s10020-025-01118-4 (PMC11841161; doi:10.1186/s10020-025-01118-4)
Supplement: Supplementary file 1 — Supplementary Material 1 [file 10020_2025_1118_MOESM1_ESM.docx]

**Gαq/11 aggravates acute lung injury in mice by promoting endoplasmic reticulum stress-mediated NETosis**

**Supplementary material**

**Supplementary Material and Method**

**Bone marrow-derived macrophages (BMDMs) isolation**

Macrophages were harvested from the bone marrows of C57BL/6J WT mice according to previously published procedures (Weischenfeldt & Porse, 2008). In brief, bone marrow cells were collected from the femurs and tibias of mice by flushing with Dulbecco's modified Eagle's medium (DMEM). The red blood cells were lysed with RBC lysis buffer (Thermo Fisher Scientific) for 10 min. The bone-marrow cell suspension was cultured in DMEM supplemented with 10% fetal bovine serum, 50 μg/mL penicillin/streptomycin and 10 ng/mL recombinant mouse macrophage colony stimulating factor (M-CSF) (Sigma–Aldrich). At day 3, comparable volume of fresh DMEM was added to the culture. At day 5, the culture medium was changed. At day 7, the cells were well differentiated and ready for use in the subsequent experiments.

**Macrophage and neutrophil count in BALF**

Flow cytometry was performed to identify and count macrophage and neutrophil in BALF. Single cells were gated on FSC-A/FSC-H. Live cells were gated on Zombie^-^/FSC-H. Leukocytes were gated on CD11b+/FSC-H. The identification of neutrophils was gated on CD11b^+^Ly6G^+^ and macrophage was gated on CD11b^+^F4/80^+^.

**Supplementary Figures and Legends**


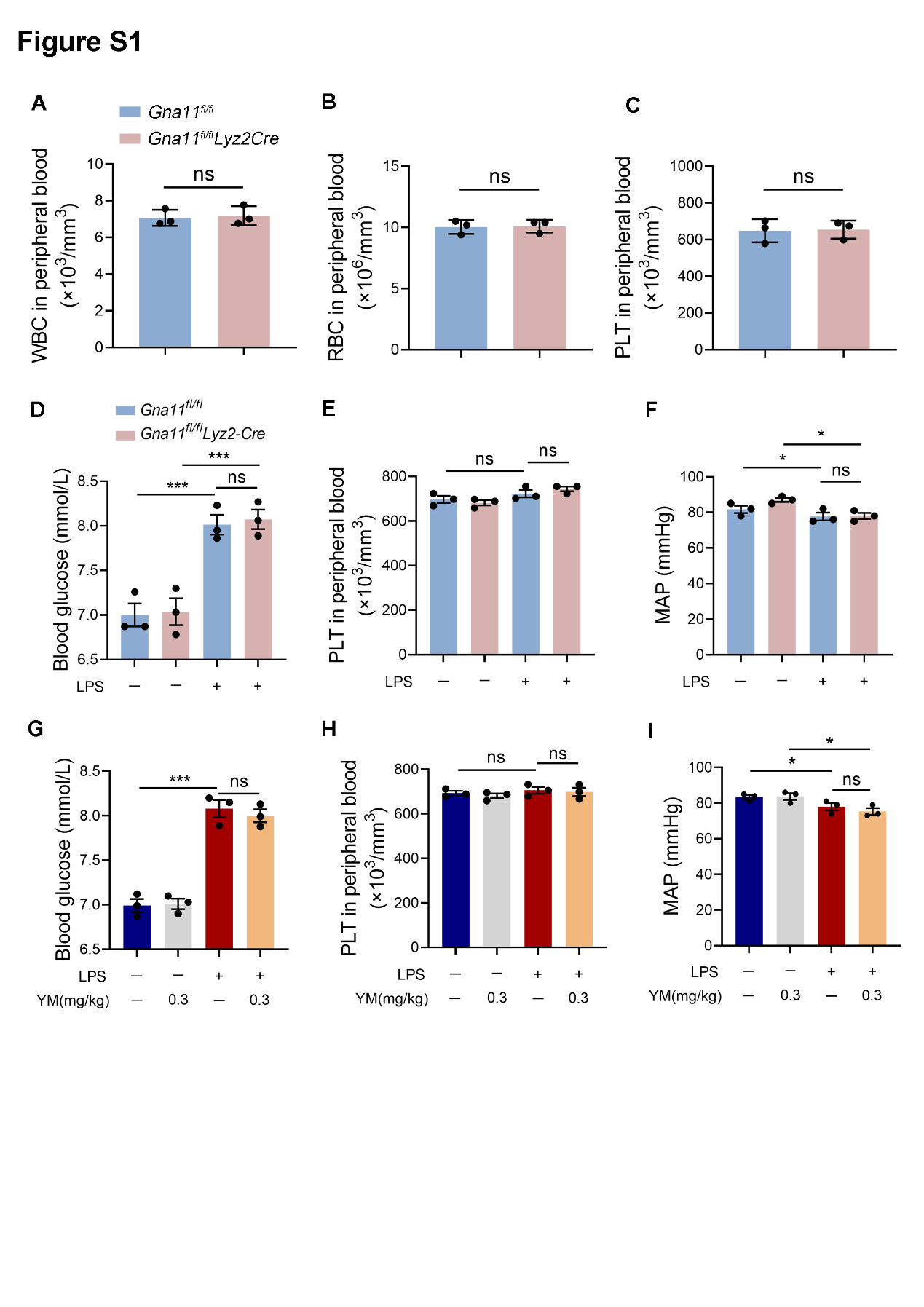


**Fig. S1**

**(A)** The numbers of white blood cell (WBC) in peripheral blood of mice. **(B)** The numbers of red blood cell (RBC) in peripheral blood of mice. **(C)** The numbers of platelet (PLT) in peripheral blood of mice. **(D-F)** *Gna11^fl/fl^* and *Gna11^fl/fl^Lyz2Cre* mice received an orotracheal atomizing injection of LPS (10 mg/kg) for 24 hours. **(D)** Blood glucose levels of mice. **(E)** The numbers of PLT in peripheral blood of mice. **(F)** Mean arterial pressure (MAP) of mice. **(G-I)** YM254890 was administered to mice 3 hours post-LPS stimulation and sampled at 24 hours. **(G)** Blood glucose levels of mice. **(H)** The numbers of PLT in peripheral blood of mice. **(I)** MAP of mice. The results shown were representative of three independent experiments. Data are expressed as the mean ± SEM. **P* <0.05; ***P* <0.01; ****P* <0.001 as indicated.


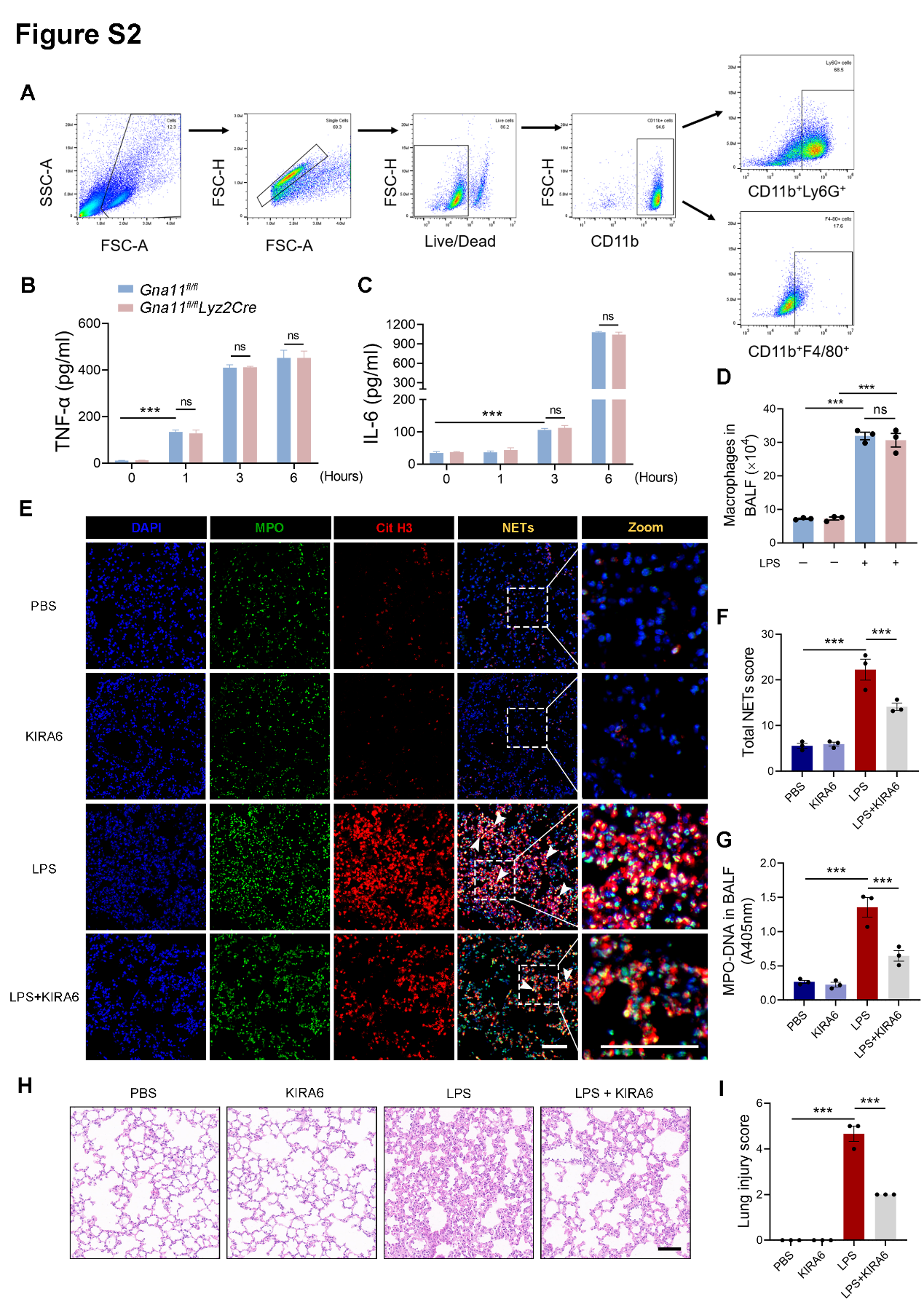


**Fig. S2.**

**(A)** The gating strategies for flow cytometry. Single cells were gated on FSC-A/FSC-H. Live cells were gated on Zombie^-^/FSC-H. Leukocytes were gated on CD11b^+^/FSC-H. The identification of neutrophils was gated on CD11b^+^Ly6G^+^ and macrophage was gated on CD11b^+^F4/80^+^. **(B-C)** Macrophages from *Gna11^fl/fl^* and *Gna11^fl/fl^Lyz2Cre* mice were stimulated by 1 μg/mL LPS for different timepoints. **(B)** TNF-α in supernatant. **(C)** IL-6 in supernatant. **(D)** *Gna11^fl/fl^* and *Gna11^fl/fl^Lyz2Cre* mice received an orotracheal atomizing injection of LPS (10 mg/kg) for 48 hours. The numbers of macrophages in BALF. **(E-I)** Mice were pretreated with KIRA6 (5mg/kg, intraperitoneal injection) for 1h and then received an orotracheal atomizing injection of LPS (10 mg/kg) for 24 hours. **(E)** Representative immunofluorescence staining of NETs in the lung tissues of mice (DAPI: blue; MPO: green; Cit H3: red; Merge: Cit H3 **^+^** MPO **^+^** NETs; scale bars, 100 μm). **(F)** Total NETs score of respective lung section was calculated. **(G)** MPO-DNA complexes in BALF. **(H)** Representative H&E staining of the lungs (scale bars, 200 µm). **(I)** Lung injury score of sections. The results shown were representative of three independent experiments. Data are expressed as the mean ± SEM. **P* <0.05; ***P* <0.01; ****P* <0.001 as indicated.

**Reference**

Weischenfeldt J, Porse B (2008) Bone Marrow-Derived Macrophages (BMM): Isolation and Applications. *CSH Protoc* 2008: pdb.prot5080
